# Community-Based Care Ecosystem: Improving Outcomes for People with Dementia and Their Caregivers

**DOI:** 10.1093/geroni/igaf122.2573

**Published:** 2025-12-31

**Authors:** Jill Cigliana, Kelli Barton, Jeriel Bohall

**Affiliations:** Memory Care Home Solutions, St. Louis, Missouri, United States; University of Missouri-Kansas City, Kansas City, Missouri, United States; University of Missouri-Kansas City, Kansas City, Missouri, United States

## Abstract

The impacts of dementia diagnoses can be far-reaching, from state and federal financial burdens to strain on healthcare systems. People living with dementia (PWDs) are often cared for at home by family and friends; however, they may experience increased rates of adverse health events, higher utilization of healthcare services, and have limited access to individualized behavioral interventions. Despite overwhelming evidence that non-drug approaches are the gold standard of care, dementia programs rarely exist outside of academic trials. Care Ecosystem (CE) is one model of dementia care designed to provide personalized, cost-efficient care for people with dementia and their caregivers. A community-based dementia services organization implemented a state-wide adaptation of the CE model in Spring 2023 and used longitudinal data to measure its impact on PWDs and their caregivers. Over a 6-month enrollment phase, outcomes showed a statistically significant increase in PWD quality of life (p = .002, n = 112), measured by proxy ratings of the modified Dementia Quality of Life (DEMQOL) scale. Additionally, PWD risk for hospital admission decreased from baseline to 6 months (p = .007, n = 94), as measured by the modified LACE index. Caregiver self-efficacy was also measured across 4 items, and by 6 months, caregivers reported greater confidence getting needed services, finding help, managing caregiving challenges, and managing PWD behavioral changes (p < .001, n = 130). Feedback from semi-structured partner interviews also revealed broader benefits of CE implementation, including strengthened community partnerships, staff readiness, and organizational growth.

